# A Comprehensive View of the Optimization of Chromium (VI) Processing through the Application of Electrocoagulation Using a Pair of Steel Electrodes

**DOI:** 10.3390/ma16083027

**Published:** 2023-04-11

**Authors:** Ľubomír Pikna, Mária Heželová, Dagmar Remeteiová, Silvia Ružičková, Róbert Findorák, Jaroslav Briančin

**Affiliations:** 1 Faculty of Materials, Metallurgy and Recycling, Technical University of Kosice, Letná 9, 042 00 Kosice, Slovakia; 2 Institute of Geotechnics, Slovak Academy of Sciences, Watsonova 45, 040 01 Kosice, Slovakia

**Keywords:** chromium, electrocoagulation, metal recovery, secondary raw materials, optimization

## Abstract

In the presented article, an electrocoagulation method using a steel cathode and a steel anode was used to obtain chromium from laboratory-prepared model solutions with known compositions. The study aimed to analyze the effect of solution conductivity, pH, and 100% efficiency of chromium removal from the solution, as well as the highest possible Cr/Fe ratio in the final solid product throughout the process of electrocoagulation. Different concentrations of chromium (VI) (100, 1000, and 2500 mg/L) and different pH values (4.5, 6, and 8) were investigated. Various solution conductivities were provided by the addition of 1000, 2000, and 3000 mg/L of NaCl to the studied solutions. Chromium removal efficiency equal to 100% was achieved for all studied model solutions for different experiment times, depending on the selected current intensity. The final solid product contained up to 15% chromium in the form of mixed FeCr hydroxides obtained under optimal experimental conditions: pH = 6, I = 0.1 A, and c (NaCl) = 3000 mg/L. The experiment indicated the advisability of using a pulsed change of electrode polarity, which led to a reduction in the time of the electrocoagulation process. The results may help in the rapid adjustment of the conditions for further electrocoagulation experiments, and they can be used as the optimization experimental matrix.

## 1. Introduction

The steel industry not only produces various types of steel but also produces a large amount of slag and other waste. According to the type of steel produced, slag can contain up to 5% chromium. Therefore, slag can serve as a source of chromium and other metals, once the recovery methods are ecological and of a reasonable cost. Hydrometallurgically obtained slag leachates can be processed by the electrocoagulation method (EC). EC essentially represents in situ generation of a coagulation agent using appropriate electrodes and an electric current. The principle of the method is generally available in numerous areas of the available electrochemical literature and its implementation depends on the type of compound to be removed and the pair of electrodes used. EC is suitable for recovering a variety of metals and compounds from leachate or wastewater.

Copper, nickel [1], fluoride [2], chromium [1,3], various organic pollutants [4,5,6], phosphorus [6], boron [7], nickel, lead, mercury, zinc, arsenic, cadmium, and chromium [8] are some of the components that can be treated or removed from dyehouse or distillery wastewaters, leachate of environmental samples, leachate of industrial waste, or underground water using EC with a combination of different working electrodes.

The effect of anode and cathode materials on metal ions has been confirmed by many papers [9,10,11]. The degree of efficiency of the EC process is different for each metal when changing the pair of electrodes. Studies on alternative foam types of electrodes or electrodes made from metal chips have also been published, and in one study, the efficiency of the EC method on greywater was 93% and 87% compared to the 84% efficiency of a traditional aluminum plate electrode [12].

In an effort to reduce the amount of chemicals used in the processing of various raw materials or in chemical analysis, the question arises as to whether it is not more appropriate to use electrocoagulation instead of the direct application of various chemical coagulation agents for water decontamination. Ackbal et al. focused, in their article, on a comparison of the EC method and “conventional” methods in the removal of heavy metals (Cu, Cr, and Ni) [1]. As a result, EC proved to be an equally effective and ultimately cheaper method with lower chemical consumption. Hybrid types of electrocoagulation such as photo-, peroxi-, and photo-peroxi-electrocoagulation with the use of a Fe electrode proved to be a possible modification of the process with promising results in the removal of organic pollutants from distillery industrial effluent [9].

For the remediation of tannery wastewater, the combination of EC with other pre-treatment methods was discussed by Verma et al. [13]. Among them, ultrasonication, adsorption, biological treatment, and membrane processes were used to improve EC performance. The use of an additional method is associated not only with advantages but may be associated with an excessive amount of energy used, the formation of by-products, and the required UV energy for a long period.

Many authors have focused on its application for the removal of trivalent or hexavalent chromium from wastewater solutions of any type. Al-Qodah et al., in their review [8], provide a detailed overview of works focused on EC for heavy metals removal (Cr(III), Cr(VI), Cu, Cd, Zn, Ni, As(III), Pb, and Hg) under different experimental conditions with Fe, Al, Cu, stainless steel, or Zn electrodes and its combinations. The Cr(III) or Cr(VI) concentrations in the reviewed studies were from 10 mg/L to 2235 mg/L (with most of them being under 500 mg/L) with removal efficiencies from 42% to 100% or 7.4% for the highest-mentioned concentration.

The authors of studies usually monitor the effectiveness of the decontamination process in their chosen time frame, often with high current densities, focusing on the removal of the contaminant, regardless of the composition of the solid product or the intensity of the electric current used, including side effects, such as heating the solution at higher current intensities.

The approach of the present study is different. The work focuses on the optimization of the process of obtaining chromium from the model solutions in the form of a further processable product and, at the same time, obtaining the decontaminated water.

Numerous variations of the experimental conditions lead to the formulation of the best conditions for combinations of current intensity, the concentration of chromium(VI) solution, conductivity, and pH value. The overall optimization matrix was compiled from the results. TGA and SEM-EDX analyses were carried out for verification of the obtained EC product. Overall, the present study is expected to help the rapid adjustment of EC experimental conditions for future laboratory and real sample analyses.

## 2. Materials and Methods

Model solutions for electrocoagulation experiments with Cr(VI) concentrations of 100, 1000, and 2500 mg/L were prepared from potassium dichromate.

A laboratory power supply EA-PS 3032-10B (ELEKTRO-AUTOMATIK), with a max output 10 A and 32 V–DC power source, a shaft stirrer WISESTIR HT50DX with rpm regulation, and a pH meter ORION 3 STAR (THERMO) were used.

Steel plates ST37-2 with active surface dimensions of 50 × 40 × 2 mm were directly dipped in the solution. The overall dimensions of the electrodes were 150 × 40 × 2 mm. The distance between the electrodes was 5 cm.

The influence of NaCl was studied for concentrations of 1000, 2000, and 3000 mg/L of NaCl.

The influence of pH was verified for the values 4.5, 6, and 8. The initial pH was adjusted at the beginning of the experiment and regulated throughout the duration of the experiment. For this purpose, 1:1 hydrochloric acid and/or 1 mol/dm^3^ NaOH solutions were used.

In the EC process, current intensities of 0.1, 0.5, 1, and 2 A (mean current densities: 4.76, 23.8, 47.6, and 95.2 mA/cm^2^, respectively) were tested.

The volume of solutions equal to 350 mL and the rotations of the shaft stirrer of 250 rpm were constant for each experiment. All of the reagents used were of analytical purity grade.

**Figure 1:**
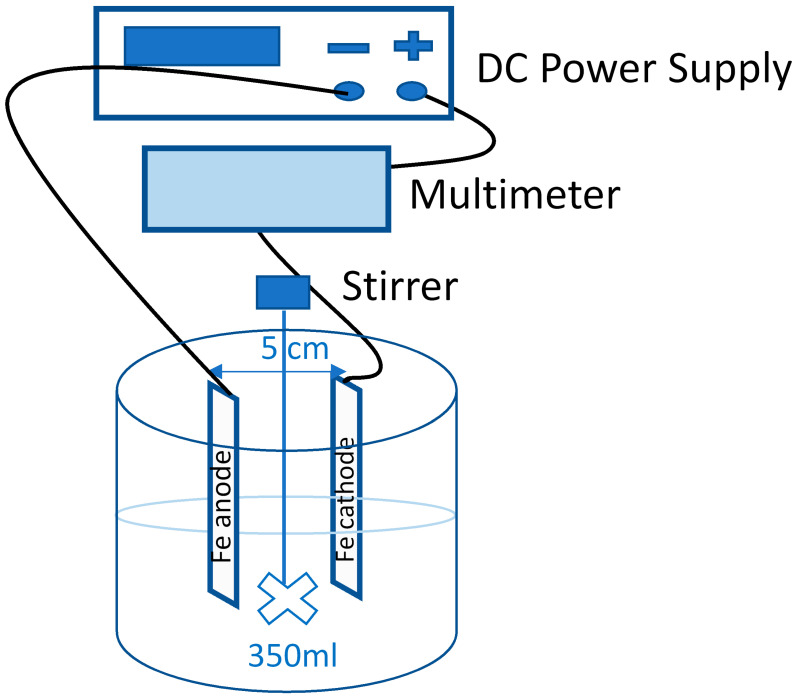
Scheme of the experimental arrangement.

The scheme of the experimental arrangement is shown in Figure 1.

The EC experiments lasted for the time period needed for the absolute removal of chromium from the solution. As a result of using the same volume of each sample and the condition of total removal of Cr from the solution, different sampling time intervals were chosen depending on the chromium concentration in the solution and the applied current intensity.

Chromium in the filtrates, as well as Cr and Fe in the solid products, was determined by atomic absorption spectrometry (VARIAN 20+); in all tables, graphs, and calculations, a concentration of 0 mg/L means that it was below the LOQ of AAS.

Solid products were characterized by TGA, SEM, and EDX analyses. The derivatograph C, MOM, was used for TGA measurements of the change in product weights of the direct EC process under heating conditions. The thermal gradient of sample heating was 10 °C/min.

A scanning electron microscope (SEM), TESCAN MIRA 3 FE, with microanalytical EDX equipment, OXFORD INSTRUMENTS, was used for EC product characterization.

The overall combination of experimented model solutions is given by rows 1–22 in Table 1. It contains the current, conductivity, concentration of NaCl and Cr(VI), and voltage (which changes during the measurement depending on the conductivity of the solution; the table shows the average voltage values).

## 3. Results and Discussion

In the process of optimization, the influence of pH, current intensity, and NaCl concentration on the overall time of total Cr removal from the solution, the energy consumption, as well as the Cr/Fe ratio in solid products, was observed. In the process of electrocoagulation by using Fe-Fe electrodes, hexavalent chromium dissolved in water was reduced in the solution by divalent iron ions produced by the dissolution of the Fe anode. The high efficiency of Cr removal by electrocoagulation using the Fe-Fe electrode pair has also been confirmed by the works of other authors [10,14,15].

Another monitored parameter was the temperature of the solution, which increased with the intensity of the current. In solutions with 1000 mg/L of chromium and 3000 mg/L of NaCl, the mean increases were 1, 5, 15, and 30 °C for I = 0.1, 0.5, 1, and 2 A, respectively.

### 3.1. Time Dependence of the EC Process

The duration of the EC process was customized to the aim of total, 100% efficiency, Cr removal. Therefore, different sampling times were also chosen.

**Figure 2:**
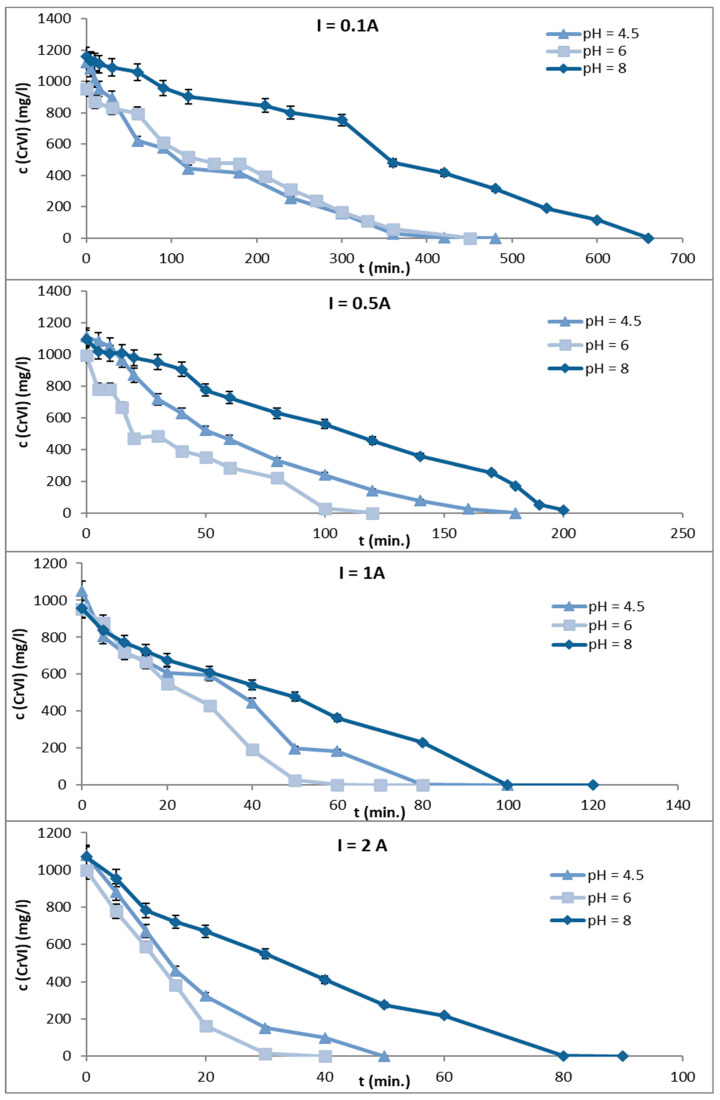
Decrease in Cr concentration in model solutions at pH 4.5, 6, and 8 for current intensities of 0.1, 0.5, 1, and 2 A.

Figure 2 shows the curves of decreasing Cr concentration during the EC process for the pH values 4.5, 6, and 8 and for the current intensities of 0.1, 0.5, 1, and 2 A. The calculated concentration of Cr was 1000 mg/L for the preparation of the model solution. Experiments were performed with a NaCl concentration equal to 3000 mg/L. Table 2 summarizes the time (in minutes) when the Cr concentration reached zero as a function of pH and amperage.

The EC process was completed earlier when the solution was kept at pH = 6 for all current intensities. A higher current intensity caused a shorter time of Cr removal, measured in tens of minutes for I = 1 A or 2 A compared to hundreds of minutes for I = 0.1 A. On the other hand, a higher current intensity was associated with an increase in solution temperature and a decrease in the Cr/Fe ratio—which is crucial for this type of Cr-containing product.

**Figure 3:**
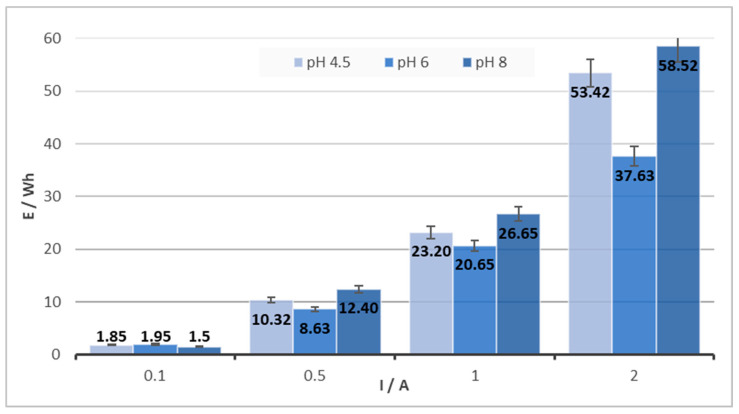
Energy consumption for the solution c(Cr(VI)) = 1000 mg/L and c(NaCl) = 3000 mg/L.

The shorter duration of the electrocoagulation process at pH = 6 is also reflected in the total consumption of electric energy (Figure 3). After converting to 1 g of solid product, the average energy consumption was 1.1, 3.58, 8.75, and 11.07 Wh for currents of 0.1, 0.5, 1, and 2 A and pH = 6. There is a significant difference in energy consumption for the lowest and highest current intensity used for all of the studied pHs. Taking into account the results obtained, it can be concluded that for the Fe-Fe electrode combination, pH = 6 is optimal, as has already been observed by other authors [16]. A chromium removal efficiency equal to 100% was also achieved at pH = 8, similar to the results described by Handman et al. [17], as well as the results reported by the authors Keshmirizadeh et al. and Dermentzis et al. [18,19] at pH = 4.5. However, the results presented here show that environments with different pHs of 4.5 to 8 are usable. The total time may be unimportant if the goal is to completely remove Cr from the solution using a small amount of energy and, at the same time, to obtain a solid product with the highest possible Cr/Fe ratio, which can be further processed. In addition, Dermentzis et al., Aguilar-Ascón et al., and Rodriguez et al. [19,20,21] describe faster removal of chromium when using a higher current, even at the expense of higher energy consumption or heating of the solution. In these works, the priority was set as 100% effectiveness of Cr removal from the solution as well as, the achievement of the highest possible Cr/Fe ratio in the solid product. The prepared solid products, with a high portion of Cr, can be used as a source of chromium for metallurgical applications, whereby the chromium, originally found in the metallurgical waste (slag), can be returned to the production process.

Prevention of electrode passivation, anode corrosion, and the liberation of Fe(II) ions into the solution, as well as improving the conductivity of the solution are the functions of NaCl’s presence in the solutions leading to successful Cr removal from the solution. In the experiments, concentrations of 1000, 2000, or 3000 mg/L of NaCl were used combined with 100, 1000, or 2500 mg/L of Cr(VI). No significant effect of NaCl concentration on the duration of the EC process to reach zero Cr concentration in the solution was observed. On the basis of the observation of the experiments, it was found that a certain concentration of NaCl (at least 1000 mg/L), especially at a concentration of 100 mg/L Cr, is necessary for sufficient conductivity of the solution and maintaining the current intensity at a certain level due to the design limitations of the voltage source used. Sufficient conductivity is also necessary due to energy consumption. Higher conductivity of the solution requires a lower voltage to attain the required current intensity and, as a result, lower electric energy consumption. While maintaining parameters such as pH and chromium concentration with a difference only in NaCl concentration, a three-fold decrease in NaCl concentration can cause a reduction in energy consumption by 20 to 50%, depending on the intensity of the current applied to the given solution.

### 3.2. The Cr/Fe Ratio Observation

Table 3 summarizes the composition of the solid electrocoagulation product connected to the monitored parameters for the experimental conditions in rows 1–22 according to Table 1.

**Figure 4:**
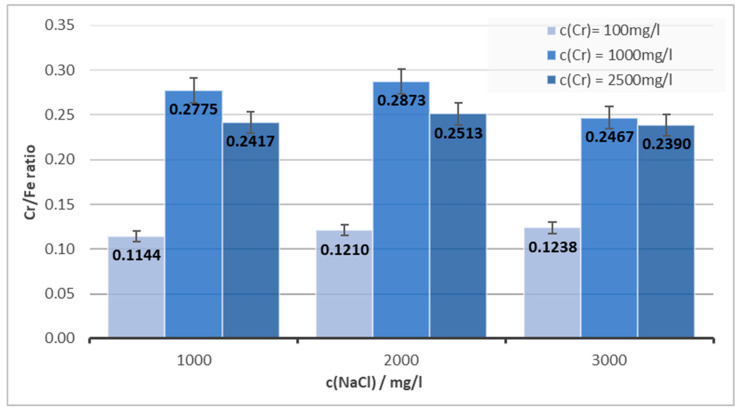
The Cr/Fe ratio’s dependence on NaCl concentration for pH = 6 and I = 1 A.

Figure 4 shows the dependence of the Cr/Fe ratio in the solid product on the concentration of NaCl in the solution. There is no evident trend in the relationship between NaCl and the Cr/Fe ratio for all the Cr concentrations studied, and the values are within the deviation interval for each concentration.

**Figure 5:**
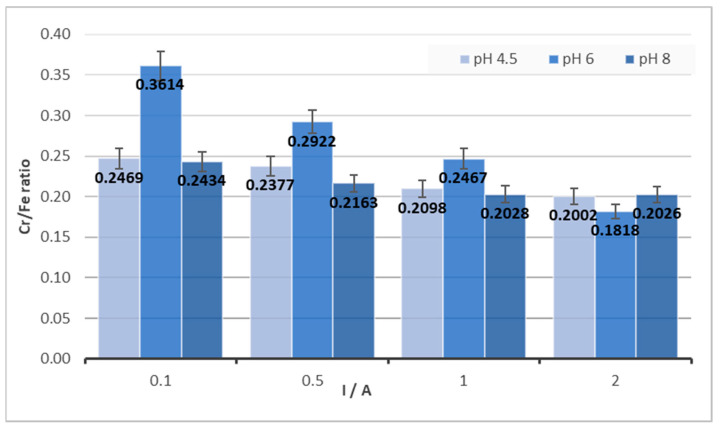
The Cr/Fe ratio’s dependence on pH for c(Cr(VI)) = 1000 mg/L and c(NaCl) = 3000 mg/L for different amperages.

Another observation is the dependence of the Cr/Fe ratio on the pH of the solution from which the product was created. As can be seen in Figure 5, for a concentration of Cr 1000 mg/and a concentration of NaCl 3000 mg/L, the pH of the solution significantly affects the composition of the final solid product and the best Cr/Fe ratio in the product is for pH = 6. At this pH, an approximately 30% percent higher Cr/Fe ratio was achieved compared to pH 4.5 or 8 at a current of 0.1. The observation was similar for currents 0.5 A and 1 A, although with a percentage difference of approximately 20% and 16%. However, when high current intensities (2 A) were used, this positive effect was lost and, as indicated by the data in Figure 5, pH did not influence the Cr/Fe ratio.

In an effort to further improve the Cr/Fe ratio in the solid product in favor of chromium, an experiment in which the polarity of the electrodes was changed at 5 min intervals was carried out. An experiment was carried out for pH = 6 and I = 0.1 A. Although in the case of constant connection, the Cr/Fe ratio was 0.3614, when the polarity of the electrodes was repeatedly changed, a value of 0.3989 was achieved. This represents an increase of 10.3%. Therefore, it is possible to suppose that low currents and a higher frequency of electrode polarity change could contribute to an increase in the proportion of Cr in the solid product.

**Figure 6:**
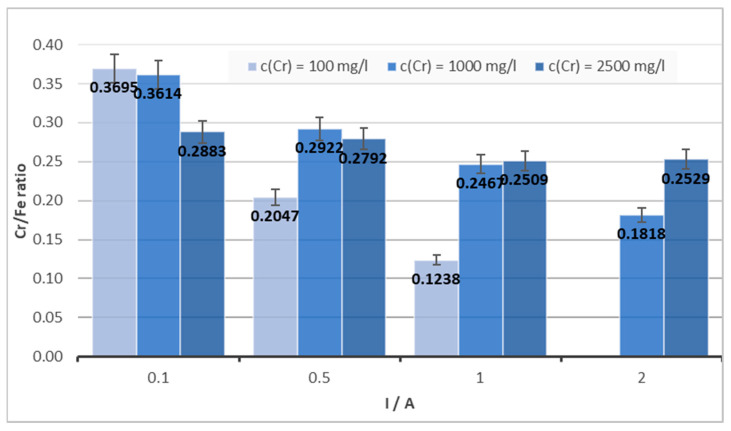
The Cr/Fe ratio’s dependence on amperage for different concentrations of Cr(VI), pH = 6, c (NaCl) = 3000 mg/L.

The dependence of the Cr/Fe ratio on amperage is depicted in Figure 6. A significant decrease in the Cr/Fe ratio for a given pH, mainly for the solution with a chromium concentration of 100 mg/L, can be observed. For a solution with a relatively low concentration of chromium (100 mg/L) and under the chosen experimental conditions described above (mainly a NaCl concentration maximum of 3000 mg/L), as well as with the hardware limitations of the DC supply source used, it was not possible to carry out the experiment at a current of 2 A mainly due to the relatively low conductivity of the solution. Application of a high current on the solution with a low chromium concentration led to the removal of all chromium present and could produce a large amount of Fe ions inside the solution within a short time. As a consequence, the Cr/Fe ratio could be unsatisfactory. For the solutions with the lowest concentration of chromium (100 mg/L), the biggest differences in the Cr/Fe ratio were observed depending on the current. On the other hand, for the highest studied concentration of 2500 mg/L, minimal differences in intensity depending on the applied current were observed.

### 3.3. Overall Experimental Matrix of the Obtained Results

On the basis of the extensive variation in the experimental conditions, an overview of the results in Table 4 was created to help with the rapid determination of the advantages and disadvantages that could influence the purpose of electrocoagulation. The + sign means suitable conditions for electrocoagulation; on the other hand, the − sign describes unsuitable conditions for achieving the desired effect.

### 3.4. Solid Product Characterization

**Figure 7:**
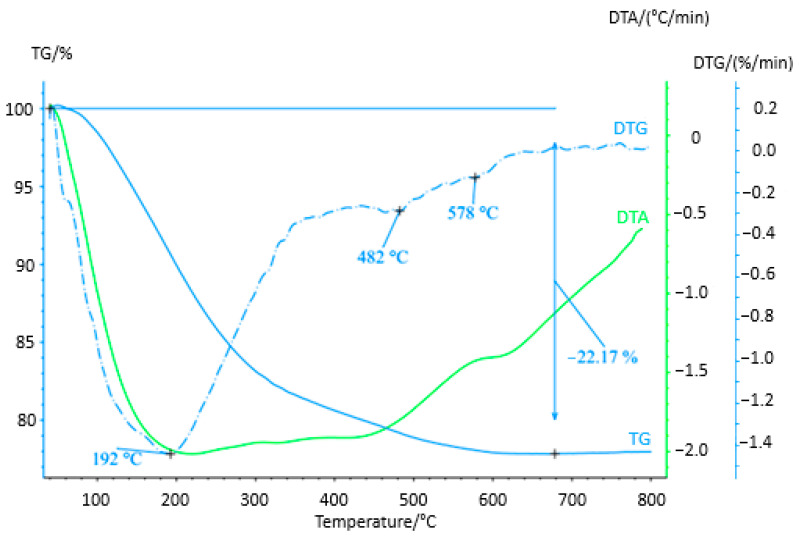
TGA analysis of the solid product of the EC process obtained from the model solution c(Cr(VI) = 2500 mg/L), c(NaCl) = 3000 mg/L, pH = 6, I = 0.1 A.

On the basis of the XRD measurement, the solid product was evaluated as amorphous, probably in the form of iron and chromium hydroxides. Therefore, thermogravimetric analysis (TGA) was performed to obtain more detailed information on the composition of the product. The aim was to find out if the product only has the expected composition in the form of hydroxides and oxides-hydroxides or if it also contains some impurities originating from the chemicals used in electrocoagulation. Establishing its behavior at elevated temperatures and establishing the temperature for further processing of the solid product to crystallin oxidic form were the goals of the study as well. The sample shows thermal instability up to 800 °C (Figure 7) with a total mass loss of approximately 22% (confirmed by repeated measurements). It is a continuous function of weight loss with a change in its rate, while in the first step, the maximum mass gradient is at a temperature of 192 °C, and the temperature interval of this change starts approximately from 100 °C to 300 °C when the peaks of the second and third steps of decomposition occur with two maxima around 482 °C and 576 °C, respectively. In the mentioned interval of 300–680 °C, there can be observed a slight decrease in mass loss. In the entire monitored interval of thermal heating, an endothermic effect is recorded, which is probably related to the release of bound hydrated water to Fe and Cr oxides, which confirmed our assumption about the composition of the solid product.

**Figure 8:**
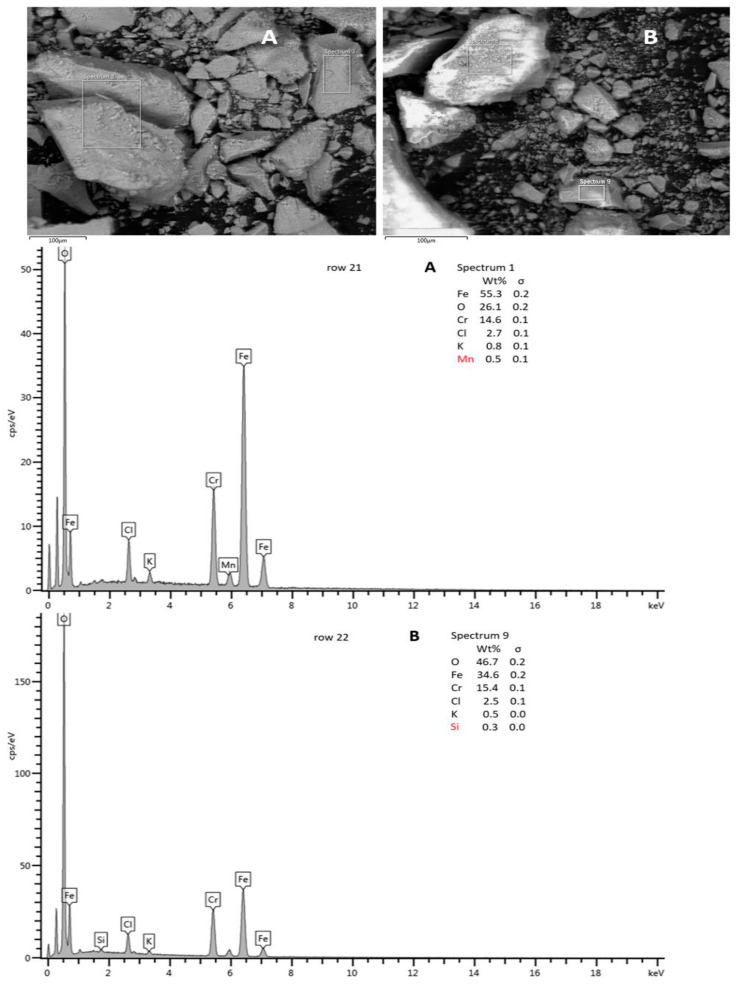
SEM scan and EDX analysis of solid product prepared under the conditions described in Table 1, row 21 (**A**) and SEM scan and EDX analysis of solid product prepared under the conditions described in Table 1, row 22 (**B**).

SEM-EDX analysis (Figure 8) was performed for the composition determination in the micro area for solid products prepared under the conditions described in Table 1, rows 21 and 22. According to the EDX spectra measured in the SEM images, marked areas of elements Fe, Cr, O, K, and Cl and under 0.5% Si, Al, or Mn were each identified. From spectrum 1 in Figure 8A (the entire area shown in the first SEM image), chromium, iron, and oxygen cover 96% of all identified elements, and at spectrum 9 in Figure 8B (small area in the second SEM image), it was 96,7%. The amounts of chromium determined by the EDX analysis were 14.6% or 15.4% for the mentioned areas, and the Cr/Fe ratios calculated from the EDX results were 0.2640 and 0.3298, respectively. However, the composition and amounts of Fe and Cr were in good agreement with those in the AAS analysis (compared with the calculated Cr/Fe ratio of AAS 0.2390 and 0.3989) mentioned in Table 3, rows 21 and 22, respectively, taking into account local microanalysis of the solid product and bulk analysis of the dissolved solid product.

## 4. Conclusions

1. Electrocoagulation with a steel cathode and a steel anode was applied to the treatment of model solutions containing chromium (VI) of 100, 1000, and 2500 mg/L. The production of an EC product with a high proportion of chromium was the important investigated parameter.
2. Based on the experimental results, it was observed that Fe-Fe electrodes pair with an applied current intensity of 0.1 A, an inter-electrode distance of 5 cm, solution pH = 6, and a NaCl concentration of 3000 mg/L are the ideal operating conditions to obtain a solid product containing up to 15% Cr. The optimal concentration of NaCl is important due to the sufficient conductivity of the solution and the associated energy consumption.
3. The duration of EC experiments increased in the order of the applied current intensities: 2 A, 1 A, 0.5, and 0.1 A. In reverse order, the ratio of Cr in the EC products decreased. The use of low currents appears suitable because of a better ratio of Cr/Fe, as well as lower energy consumption and almost no overheating of the solution during relatively long experiments with high concentrations of chromium in the solution. However, if the intention is to quickly remove the contaminant, regardless of the composition of the product, it is possible to use high currents. This is primarily the case for many environmental studies that deal with the removal of contaminants.
4. The influence of NaCl concentration on the proportion of chromium in the solid product has not been confirmed.

The experimental result matrix in Table 4 could serve as a tool for the rapid set-up of EC experiments. It is presumed to be useful for the facilitation of other analyses leading to the removal of chromium, mainly within environmental analyses.

Overall, the experimental results indicate that the EC process is able to be an effective tool for obtaining Cr-rich products. For this purpose, the serious limitations of the study are having a low or very high pH, the application of high current intensities connected with the solution overheating, and insufficient conductivity leading to high energy consumption.

The benefit of the work is the observation of an increase in the Cr/Fe ratio when the polarity of the electrodes was repeatedly changed. An increase of 10.3% was observed in this parameter. This observation opens the way to the use of pulsed electrocoagulation, the results of which can be compared with this study in the future stage of research.

From an environmental point of view, 100% efficiency in removing Cr is a useful tool for the remediation of Cr-contaminated wastewater. This is the goal of our future EC research.

## Figures and Tables

**Table 1:** Investigated model solutions experimental set.

	c (Cr VI) (mg/L)	I (A)	U (V)	c (NaCl) (mg/L)	G (mS)	pH
1.	1000	0.1	3	3000	7.2	6
2.	1000	0.5	8.5	3000	7.2	6
3.	1000	1	16	3000	7.2	6
4.	1000	2	25	3000	7.2	6
5.	1000	0.1	2.2	3000	7.2	8
6.	1000	0.5	6.7	3000	7.2	8
7.	1000	1	12.7	3000	7.2	8
8.	1000	2	19	3000	7.2	8
9.	1000	0.1	2.5	3000	7.2	4.5
10.	1000	0.5	8.5	3000	7.2	4.5
11.	1000	1	13	3000	7.2	4.5
12.	1000	2	25	3000	7.2	4.5
13.	100	0.5	22	1000	2.2	6
14.	100	0.5	15.5	2000	3.5	6
15.	100	0.5	12	3000	4.7	6
16.	1000	1	29	1000	4	6
17.	1000	1	23	2000	6	6
18.	1000	1	16	3000	7.2	6
19.	2500	1	12.5	1000	10.4	6
20.	2500	1	12.5	2000	11.2	6
21.	2500	1	10.3	3000	12.2	6
22. *	1000	0.1	3	3000	7.2	6

* The experimental conditions are the same as in row 1, but the electrode polarity was changed at 5 min intervals.

**Table 2:** Minutes until the chromium concentration was zero in the solution.

c (Cr VI) (mg/L)	pH	0.1 A	0.5 A	1 A	2 A
100	6	60	15	8.5	n/a
1000	4.5	480	180	100	50
	6	450	120	80	40
	8	660	200	120	90
2500	6	1320	420	240	135

**Table 3:** Information about the composition of the solid products obtained under different (Table 1) experimental conditions.

	Solid Product Composition		
	c (Cr) (g/kg)	c (Fe) (g/kg)	Cr/Fe Ratio
1.	138.9	384.3	0.3614
2.	107.5	367.9	0.2922
3.	95.8	388.3	0.2467
4.	79.6	437.8	0.1818
5.	84.9	349	0.2434
6.	80.5	373.3	0.2163
7.	74.3	366	0.2028
8.	77.3	381.4	0.2026
9.	91.7	371.3	0.2469
10.	89.1	374.9	0.2377
11.	75.1	357.9	0.2098
12.	75.8	378.6	0.2002
13.	52.9	506.6	0.1044
14.	72.2	537.9	0.1342
15.	83.2	406.5	0.2047
16.	102	367.6	0.2775
17.	107.7	374.9	0.2873
18.	95.8	388.3	0.2467
19.	87.1	360.6	0.2417
20.	106.6	424.3	0.2513
21.	105.4	441.1	0.2390
22. *	142.4	356.9	0.3989

* The experimental conditions are the same as in row 1, but the electrode polarity was changed at 5 min intervals.

**Table 4:** Scheme of suitable (+) and unsuitable (−) experimental conditions for the EC process of low, medium, and high concentrations of chromium solutions.

Monitored Parameter	c(Cr(VI))/mg/L	pH	G/mS	I/A			
				0.1	0.5	1	2
Duration of EC/min	100	6.0	4.7	−	+	+	n/a
	1000	4.5	7.2	−	−	+	+
		6.0		−	+	+	+
		8.0		−	−	+	+
	2500	6.0	12.2	−	−	−	+
Energy consumption/Wh	100	6.0	4.7	+	+	+	n/a
	1000	4.5	7.2	+	+	−	−
		6.0		+	+	+	−
		8.0		+	+	−	−
	2500	6.0	12.2	+	+	−	−
The Cr/Fe ratio	100	6.0	4.7	+	−	−	n/a
	1000	4.5	7.2	+	−	−	−
		6.0		+	+	+	−
		8.0		+	−	−	−
	2500	6.0	12.2	+	+	+	+

## Data Availability

Not applicable.
